# Who becomes an entrepreneur after university? Evidence from Canada

**DOI:** 10.1371/journal.pone.0308949

**Published:** 2025-01-14

**Authors:** Creso Sá, Summer Cowley, Aisha Husain

**Affiliations:** 1 Department of Leadership, Higher, and Adult Education, Ontario Institute for Studies in Education, University of Toronto, Toronto, Ontario, Canada; 2 Faculty of Business and Information Technology, Ontario Tech University, Oshawa, Canada; Federal University of Goias: Universidade Federal de Goias, BRAZIL

## Abstract

In recent decades there has been significant interest among policy makers in supporting entrepreneurship among university students, with the goal to improve labor market outcomes and contribute to the economy through venture creation. Drawing from the 2018 National Graduate Survey in Canada, our study examines who engages in entrepreneurial activity after graduation, investigating differences among demographic groups and between those who participated in entrepreneurship education on campus and those who did not participate. We find that those graduates who participated in entrepreneurship education are more likely to be self-employed and own their own business three years after graduating than the general population of university graduates. We also find differences according to gender, citizenship, and socio-economic status in entrepreneurial activity. Our results are consistent with previous studies documenting demographic disparities in entrepreneurship and provide more generalizable evidence about the relationship between entrepreneurship education and subsequent entrepreneurship.

## Introduction

In recent decades, the importance of entrepreneurial activity as a key driver of economic growth has captured the attention of scholars and policy makers [1, 2]. This growing attention to entrepreneurship has contributed to the diffusion of diverse efforts and programs on university campuses to support venture creation [3, 4]. The expectations of those supporting an entrepreneurial agenda in higher education are multifaceted, and include helping students launch their own businesses, acquire relevant skills that they can use as employees, develop an innovative mindset, and nurture a local start-up community to promote economic development [5]. In Canada, the federal and provincial governments have funded multiple programs over the past two decades to encourage university students to engage in entrepreneurial activity [6]. Behind these policy efforts is the expectation that university graduates will do better in the labor market and contribute to the economy through firm creation.

The literature describes a nuanced relationship between post-secondary educational achievement and engagement in entrepreneurial activity. If on one hand a university education can impart knowledge, skills, and social capital that can be valuable to aspiring entrepreneurs, it can also lower the incentives for entrepreneurship through professional socialization and higher earning potential through employment. Meta analyses do not find a relationship between postsecondary attainment and entry into entrepreneurship [7]. Moreover, the difference in entry into entrepreneurship between those with and without university degrees appears to vary between countries [8]. Research in Canada has suggested that there is a U-shaped relationship between higher education and entrepreneurship, with university degree attainment increasing the propensity of self-employment, while lower levels of education reduce the likelihood of self-employment [9].

This study addresses the question of who among the population of university graduates enter entrepreneurship. Previous studies have reported differences related to gender, family background, income, field of study, and immigration status [9, 10] in the likelihood of entry into entrepreneurship. In general, men who are older, who have labour market experience, who were native students, and who were business and public administration majors are more likely to become entrepreneurs. Some longitudinal studies find a positive impact of participating in entrepreneurship education while in university on venture creation and self-employment [5, 11, 12], but they usually draw from small samples circumscribed to specific institutions or programs, and/or lack control groups [3, 13–16]. Our study builds upon the relatively scarce strand of studies that draws from nationally representative datasets to trace university graduates’ entry into entrepreneurship [17–19].

Our empirical focus is Canada. In studying this national setting, we seek to inform the Canadian academic and policy debate on the entrepreneurial engagement of university graduates, considering the steady decline in entrepreneurial activity in the country and the concerted efforts to nurture entrepreneurs on university campuses [20, 21]. Our study contributes to the international literature by examining the relationship between participation in entrepreneurship education (EE) and various demographic variables on later entrepreneurial activity. While much research explores the impacts of EE, most of it investigates attitudinal outcomes such as entrepreneurial intention and entrepreneurial self-efficacy rather than actual entrepreneurial activity [22–25].

We draw from the Statistics Canada’s 2018 National Graduate Student Survey to investigate the characteristics of university graduates who entered entrepreneurship and compare them to those who became employees three years after graduation. We account for gender, race, citizenship status, participation in entrepreneurship education, field of study, socio-economic status (SES), and region of residence in our analysis. We pose the following two hypotheses:

H1: There is a positive relationship between entrepreneurship education participation and subsequent entrepreneurial activity (i.e., being self-employed or owning one’s own business).
H2: Women and minorities, international students, and non-citizens are less likely to become entrepreneurs after graduation.

Our results demonstrate a positive association between participation in entrepreneurship education and becoming self-employed or owning one’s own business. Our findings also confirm expected disparities between demographic groups in participation in entrepreneurship, show some unexpected variations among ethnic groups in different kinds of entrepreneurial activity. Overall, our study provides a longer-range picture of university graduates’ entry into entrepreneurship than previous studies in the Canadian context, documenting disparities among groups in entrepreneurial engagement.

## Literature

Universities have now for decades been viewed as critical institutions supporting innovation and economic development [26]. Not only are universities anchor institutions in their regions by generating employment, attracting talent, and having a local economic impact through their operations, but they are commonly expected to support commercial and entrepreneurial outcomes of their research and teaching [27–29]. An earlier focus on the promotion of technology transfer and research commercialization, embodied in the establishment of science parks, technology transfer offices, and business incubators, has been complemented more recently by the growing popularity of entrepreneurship education offerings to students [30].

Helping to spur entrepreneurs who found new companies in technology-based sectors is a contemporary marker of success for universities, which very commonly desire to be seen as combining academic distinction with the production of innovative graduates and economic impacts [27, 29]. Drawing from alumni surveys for select universities, scholars have argued that the contributions of science and engineering graduates to start-up formation greatly outweigh that of faculty members both in quantity and quality, which in turn represents a larger source of innovation and economic development [31]. From a policy perspective, an entrepreneurial agenda has become influential in informing policy objectives relating to universities. The idea that university students should be supported to become entrepreneurs is appealing as it addresses overlapping policy concerns with graduate employability, job creation, and economic development [6]. Government efforts to stimulate entrepreneurship among this population have multiplied across North America since the 1990s. They range from targeted funding for campus entrepreneurs to incentives for universities to create programs and facilities to support them.

The literature approaches the relationship between earning a university degree and becoming an entrepreneur from different perspectives. First, more broadly, economists investigate the relationship between education and entry into entrepreneurship. As mentioned above, this is not a straightforward relationship. The broader human and social capital that university students acquire can arguably be helpful to entrepreneurial activity, but it can also open up other attractive avenues for career progression without the risks involved in launching a venture. Systematic analyses do not find an association between graduating from university and becoming an entrepreneur [7]. This relationship may of course vary between countries with different opportunity structures for university graduates; others [9] found university graduates have a greater likelihood of self-employment in Canada.

A second perspective comes from studies of the relationship between the acquisition of more specific human and social capital related to entrepreneurship among university students and entrepreneurial outcomes. Most often, scholars have investigated the effects of entrepreneurship courses and programs on students’ intentions to become entrepreneurs in the short run. Such studies usually rely on surveys where students report their entrepreneurial intentions before, during, and immediately after completing courses or graduating [32–34]. The relative abundance of this type of research follows from the convenience of ready access to entrepreneurship students to scholars specializing in and teaching this subject [35, 36]. An attempt to provide a more comprehensive look at the issue is the Global University Entrepreneurial Students Survey (GUESS), which surveys university students who have participated in entrepreneurship education in 26 countries about their entrepreneurial intent [35]. However, the implementation of the survey in the participating countries is variable in methodological and procedural terms. Overall, there is limited research tracking longer-term effects of entrepreneurship education beyond attitudinal and motivational outcomes, including the tracing of graduates’ actual engagement in entrepreneurial activity [37]. Conclusions regarding the association between entrepreneurship learning and outcomes such as venture creation have varied. While some studies suggest a positive relationship, most of these studies examine single institutions and small-scale programs [38–40] or rely on small sample sizes [31, 41, 42] making generalization of their findings difficult.

Research on this problem involves greater effort in tracing the professional trajectories of students over time and delimiting appropriate comparison groups, and very few studies explore this relationship, particularly in Canada. Recently, Breznitz and Zhang [38] analyzed two alumni surveys conducted at the University of Toronto and found a positive relationship between entrepreneurship education and firm creation. Other studies of entrepreneurship education have focused on specific programs or have limited the analysis to participants in entrepreneurship education without considering a control group [13].

Meanwhile, further literature has focused on the demographic patterns of university graduates who enter entrepreneurship [9]. Given the significance of such demographic characteristics, we included variables that spoke to each in our statistical modeling.

Gender has been found to be significant to entry to entrepreneurship, with men university graduates more likely to become entrepreneurs after graduation [9, 10, 43]. The literature finds that men develop expected outcomes at higher rates than women. Studies investigating the relationship between entrepreneurship education and venture creation, for example, have found that men become founders at higher rates than women [44, 45]. With regards to other demographic characteristics, Lyons and Zhang [11] found that members of visible minority groups experience a greater increase in likelihood of starting their own business post entrepreneurship education than did their counterparts. Amongst university graduates overall, women have been the majority over men since at least the 1990s [46]. Racial background of graduates varies across Canada and the US, but First Nations, Inuit, and Métis students are under-represented.

Finally, in terms of regional differences, the highest number of graduates in Canada can be found in Ontario and Québec (which have both the highest populations and largest number of higher education institutions in the country). Conversely, Yukon, the Northwest Territories, and Nunavut have the lowest. Within Canada’s provinces and territories, economic conditions also vary; natural resources such as forestry and oil/gas drive the economies of British Columbia, Alberta, and Saskatchewan, while mining is prevalent in the North, hydroelectric power in Quebec, and fishery/ocean industry in the Atlantic regions. In Ontario, industries such as telecommunications and manufacturing are more salient.

## Methods

This study analyzes cross-sectional data from the Public Use Microdata File (PUMF) of the 2018 National Graduates Survey (NGS), which follows the 2015 cohort of graduates three years after graduation and provides some insight into graduates’ postgraduation occupational outcomes. Permission to access the data was obtained through the Statistics Canada Research Data Centres application process. Access and analysis of the data was performed at the Toronto Research Data Centre and followed Statistics Canada’s data security and confidentiality requirements.

The target population of the 2018 NGS includes graduates living in Canada who completed their program in 2015. The survey excludes graduates from private postsecondary institutions, graduates who completed continuing-education programs at colleges and universities unless it led to a diploma or degree, graduates in apprenticeship programs and graduates living outside of Canada at the time of the survey.

### Variables

#### Variables

As the overall focus of the entrepreneurship agenda is on science and technology [40]; we have used that group as the reference category in Major of study to see whether students from those fields do indeed form businesses at higher rates. Due to similar differences in proportions, we have chosen references in the other categories to be men (gender), White (race), Canadian citizen (citizenship), did not apply for student loans (student loan application), and Ontario (region of residence).

Variables included for analysis include EE participation, self-employment status, business owner status, student loan applicant status, citizenship, race, gender, discipline of study, and region of residence (Tables 1–3).

**Table 1:** Entrepreneurship activities.

Characteristic	Yes	No	Missing
EE participation	5,178	30,068	513
Self-employed last week	1,968	28,843	4,948
Business owner	672	1,291	33,796

**Table 2:** Descriptive statistics.

Demographic Characteristic	*n*
**Applied for govt’ student loan** ^**1**^	
yes	17,797
no	17,932
missing	30
**Canadian citizen**	
yes	30,470
no	5,215
missing	74
**Gender**	
male	14,697
female	21,062
**Race**	
White	24,837
Indigenous peoples	1,491
Asian	5,373
Black	1,238
other	1,054
missing	1,766
**Discipline of study**	
STEM	9,005
Arts/Social sciences	15,265
Business	5,882
Law/medicine	5,507
**Region of residence**	
Ontario	7,254
Atlantic	7,222
Québec	6,087
West & North	15,088
missing	108

^1^ Although the NGS data did not allow for analysis of family income, we considered student loan application as a proxy for socioeconomic status.

**Table 3:** Cross-tabulation of entrepreneurship education participation and other characteristics.

	EE participation			
Self-employed last week	Yes	No	Missing	Total
Employee	3,994	24,444	405	28,843
self-employed	624	1,319	25	1,968
missing	560	4,305	83	4,948
Total	5,178	30,068	513	35,759
**Business owner**	yes	no/missing		Total
Yes	292	380		672
No	330	961		1,291
missing	4,556	29,240		33,796
Total	5,178	30,581		35,759
**Major**	yes	no	missing	Total
STEM	1,106	7,706	193	9,005
Arts/social sciences	1,468	13,714	183	15,365
business	2,255	3,535	92	5,882
law/medicine	349	5,113	45	5,507
Total	5,178	30,068	513	35,759
**Gender**	yes	no	missing	Total
Male	2,771	11,657	269	14,697
Female	2,407	18,411	244	21,062
Total	5,178	30,068	513	35,759
**Race**	yes	no	missing	Total
White	3,365	21,170	302	24,837
Indigenous peoples	198	1,271	22	1,491
Asian	886	4,381	106	5,373
Black	239	971	28	1,238
Other	192	843	19	1,054
missing	298	1,432	36	1,766
Total	5,178	30,068	513	35,759
**Region of residence**	yes	no	missing	Total
Ontario	1,148	5,989	117	7,254
Atlantic	1,074	6,039	109	7,222
Quebec	759	5,244	84	6,087
West/North/missing	2,197	12,796	203	15,196
Total	5,178	30,068	513	35,759
**Applied to government student loan**	yes	no	missing	Total
yes	2,386	15,170	241	17,797
no/missing	2,792	14,898	272	17,962
Total	5,178	30,068	513	35,759
**Canadian citizen?**	Yes	no	missing	Total
no	916	4,167	132	5,215
yes/missing	4,262	25,901	381	30,544
Total	5,178	30,068	513	35,759

NGS responses were collected online by respondent self-completion as well as a computer-assisted telephone interview method. There were a total of 19,564 respondents in the 2018 NGS PUMF. The overall response rate to the survey is 63 percent. The survey, which used a stratified simple random sample design to randomly select potential respondents within each province and territory and at four levels of education (college, undergraduate, master’s and doctorate), sampled graduates from postsecondary education institutions (such as universities, colleges, and trade schools) in Canada who graduated with degrees, diplomas or certificates in 2015.

Several new questions were introduced to the 2018 NGS to address new data needs, including a series of questions relating to entrepreneurship. The NGS includes demographic details about self-employed respondents such as race, ethnicity, citizenship and application for student loans, all of which have not been discussed in detail in previous research. The questions of interest to this study focused on academic paths, funding for postsecondary education, including government-sponsored student loans, and transition into the labour market. We selected variables that allowed us to compare the engagement in entrepreneurial activity post-graduation of students who participated in entrepreneurship education to those who did not.

Specifically, “participation in entrepreneurship education” is a variable based on student responses in the National Graduate Survey questionnaire. A value of “1” for this variable indicates that participants participation in on or more of multiple entrepreneurial activities listed in the survey questionnaire (e.g., entrepreneurship courses, business plan/pitch competition, visit entrepreneurship centre). We measured graduates’ entrepreneurial engagement using the question about whether graduates are self-employed and whether they own a business. (The latter includes the subset of respondents who said “Yes” were then asked if they own their own business after marking “Yes” to being self-employed). So, business owners in the data and in our study are respondents who self-identified as both self-employed and business owners, while self-employed respondents did not indicate that they owned a business.

We also accounted for respondents’ field of study, region of residence, and demographics (Table 4). Our selection of variables is informed by previous findings on demographics-specific effects related to the outcomes of entrepreneurship education [5, 31, 34, 47] as well as findings on the impact of academic majors [31, 48], and the significance of citizenship status to entrepreneurial activities [49]. Moreover, Green *et al*. [50] found immigrants who have lived in Canada for more than a decade had a higher propensity to own businesses than the Canadian-born population.

**Table 4:** Variables included in the model.

Variable	Description
*Dependent variables*	
selfemp	Whether respondent is self-employed at time of survey
ownbiz	Whether respondents own their own business at time of survey
*Independent variables*	
eepart	Whether respondent participated in entrepreneurial education
race	Racial background of the respondent
gender	Gender of the respondent
citizen	Whether respondent was a Canadian citizen at the time of the survey
*Control variables*	
stuloans	Whether respondent applied for government student loans
major	Respondent’s major in their 2015 academic program
region	Respondent’s region of residence in Canada at the time of the survey

Similar to recent studies of post-graduate entrepreneurship [51–53], we employed a logistic regression model in the analysis because of its appropriateness of use for variables with two possible values; in this case, whether respondents answered “yes” or “no” to a question about their status as self-employed or as business owners.

## Findings

Our sample includes 35,759 university graduates, 5.5% of whom became entrepreneurs via either self-employment, or business ownership (1.9%). Overall, our descriptive statistics show that entrepreneurship education participants tend, overwhelmingly, to be White or Asian, and are somewhat more likely to be men than women. We found significant relationships between entrepreneurial outcomes and demographic and other background characteristics.

Our findings support our first hypothesis (Tables 5 and 6). Graduates who participated in entrepreneurship education were more likely to be self-employed (1.038, p>0.001) and own their own businesses (0.585, p<0.001) three years after graduation than the overall population of university graduates. We found that individual characteristics had statistically significant relationships to self-employment and business ownership, bringing to light the differing impact of entrepreneurship education on post-graduate entrepreneurial outcomes for graduates from different backgrounds and disciplines.

**Table 5:** Model outputs, Hypothesis 1.

DV: Business Ownership	Coef.	Std. Err	Z	P>|z|	[95% Conf.	Interval]
Yes, participated in EE	0.58585	0.127144	4.61	0.000***	0.3366522	0.8350476
Is a citizen	-0.49859	0.200777	-2.5	0.013**	-0.892104	-0.105073
Race (white = reference)						
Indigenous	-0.24941	0.375208	-0.7	0.506	-0.984801	0.4859895
Asian	0.439319	0.166169	2.64	0.008***	0.1136348	0.7650036
Black	0.405096	0.354109	1.14	0.253	-0.288945	1.099138
Other	0.277352	0.333203	0.83	0.405	-0.375713	0.9304172
Sex (male is reference)	-0.85064	0.122569	-6.9	0.000***	-1.090873	-0.610412
Highest parental education						
High School	0.106969	0.271538	0.39	0.694	-0.425236	0.6391732
More than HS, less than bachelor	-0.18254	0.250622	-0.7	0.466	-0.673748	0.3086711
Bachelor’s	-0.20892	0.250038	-0.8	0.403	-0.698988	0.2811441
Graduate degree	-0.10848	0.257559	-0.4	0.674	-0.613283	0.3963296
Applied for student loan	0.283685	0.121603	2.33	0.02**	0.0453481	0.522021
Major (science, tech, eng is reference)						
Medicine (inc. residents)	0.714261	0.255634	2.79	0.005***	0.2132273	1.215294
Law	0.019295	0.375468	0.05	0.959	-0.716608	0.7551982
Business	0.719493	0.177835	4.05	0.000***	0.3709437	1.068042
Other Arts	-1.29963	0.227135	-5.7	0.000***	-1.744807	-0.854454
Other healthcare	-0.55998	0.227804	-2.5	0.014**	-1.006468	-0.113493
Social sciences	-0.3611	0.167855	-2.2	0.031**	-0.690094	-0.032114
Other	-0.1245	0.466691	-0.3	0.790	-1.039198	0.7901986

**Table 6:** Output for Hypothesis 1a.

DV: self-employed	Coef.	Std. Err.	z	P>|z|	[95% Conf.	Interval]
Self-employed						
Participated in EE	1.037928	0.083	12.58	0.000***	0.8762045	1.199652
Citizen	0.3162985	0.132	2.39	0.017**	0.0573002	0.5752968
Race (white = reference)						
Indigenous	0.0081373	0.172	0.05	0.962	-0.329242	0.3455167
Asian	0.0508765	0.113	0.45	0.652	-0.1701919	0.2719448
Black	-0.808362	0.266	-3.04	0.002***	-1.329923	-0.2868007
Other	0.1564883	0.19	0.82	0.411	-0.2168537	0.5298304
Sex (male is reference)	-0.1922743	0.074	-2.59	0.01***	-0.3376091	-0.0469394
Highest parental education						
(none = reference)						
High School	-0.4215906	0.176	-2.39	0.017**	-0.7670229	-0.0761584
More than HS, less than bachelor	-0.3795964	0.16	-2.37	0.018**	-0.6929389	-0.066254
Bachelor’s	-0.3115886	0.159	-1.96	0.05*	-0.623789	0.0006117
Graduate degree	0.0311036	0.162	0.19	0.848	-0.2866901	0.3488973
Applied for student loan	0.1084665	0.071	1.52	0.128	-0.0313774	0.2483105
Region of residence Ontario = reference)						
Atlantic	-0.8030155	0.124	-6.48	0.000***	-1.045759	-0.5602721
Québec	0.3343735	0.101	3.31	0.001***	0.1363767	0.5323704
Western	-0.2752423	0.089	-3.08	0.002***	-0.4502905	-0.1001941
Major (Science, tech, eng is reference)						
Medicine (inc. residents)	2.295	0.207	11.1	0.000***	1.889474	2.700262
Law	0.625	0.303	2.06	0.039**	0.0300262	1.219003
Business	-0.2	0.121	-1.66	0.097	-0.4368766	0.0362042
Other Arts	0.981	0.112	8.8	0.000***	0.7624729	1.199586
Other healthcare	0.299	0.135	2.21	0.027**	0.0333881	0.5636313
Social sciences	0.372	0.099	3.74	0.000***	0.1767924	0.566772
Other	0.329	0.258	1.27	0.203	-0.177254	0.8350389

In terms of our second hypothesis, we found that women were less likely to be self-employed than men (-0.192 in log odds, p = 0.010). We also found that Black respondents were less likely to be self-employed than White graduates (-0.808 in log odds, p = 0.002). Finally. we found that Canadian citizens were more likely to be self-employed than non-citizens (0.316, p = 0.017). Our findings are consistent with existing studies on gender and self-employment in Canada, which show that among the self-employed, 62 percent are men and 38 percent are women [44]. They also echo recent findings regarding the relatively lower rate of self-employment among the Black Canadians who are employed (c. nine percent) as compared to non-visible minority Canadians (c. 13 percent) [51].

In line with our expectations, participation in entrepreneurial education was associated with a greater likelihood of entrepreneurial activity. Among all respondents, those graduates who participated in entrepreneurial learning were more likely to be self-employed than the general graduate population (1.038, p>0.001), when accounting for all other variables in the model.

## Discussion

Our study contributes to scholarly and policy debate on the prevalence of entrepreneurial activity among university graduates, as one of the contributions of universities to economic development [5, 12, 38, 39]. Overall, our study provides nationally representative results on the entry into entrepreneurship of university graduates in Canada, showing that students who participate in entrepreneurship education during their studies were more likely to become entrepreneurs. It also supports previous research demonstrating asymmetries between demographic groups in entry into entrepreneurship.

While it appears that the profile of entrepreneurs amongst Canadian university graduates mirrors that broader population of self-employed and business owners, it also indicates some variation. Our findings align with research in the field documenting the greater representation of men amongst business owners across Canada and internationally [54, 55], as well as the large number of white and Asian entrepreneurs in North America [56]. Amongst self-employed graduates, women are less likely to own their own business and Asians are more likely to own their own business when compared to their White counterparts. Among all university graduates, Black graduates are less likely to be self-employed than White graduates. This is consistent with recent research showing that Black Canadians make up a smaller share of self-employed individuals than their counterparts [57].

The patterns identified in our findings are consistent with national survey results showing that most small and medium sized enterprises in Canada are male owned (69 percent), with only 17 percent being majority female owned [58]. Similarly, the higher proportion of self-employed male graduates as compared to their female peers indicates a similar pattern of female under-representation among the university graduate population as that shown in research indicating that women made up about 38 percent of self-employed workers across Canada [59]. However, with respect to ethnicity, our findings are different than those of the general population. In contrast to our study’s results on immigrants, Picot and Ostrovsky [60] have found that both immigrants and Canadians with immigrant parents (second generation) have higher rates of business ownership than Canadians with Canadian born parents (third or greater generations).

In addition, our findings highlight the segmentation of different demographic groups in pursuing entrepreneurship as a career option after attending university. In terms of the expectation that entrepreneurship education students are more likely to go on to become entrepreneurs, our findings are more conclusive than previous studies, albeit consistent with previous results. Whereas much of the literature has shown a positive relationship between participation in entrepreneurial education and subsequent venture creation and self-employment, many of these studies were highly localized and small scale [33]. Our study, in contrast, draws from a much larger, nationally representative sample and provides a longer-term view on the outcomes of campus entrepreneurial experiences.

The patterns we identify among Canadian university graduates are generally different than those of other studies drawing from national-level data to examine entrepreneurial behavior [18, 19, 58, 61, 62]. Previous studies found that entrepreneurship post-university was not significantly more or less likely for university graduates versus dropouts in Denmark [61], and that timing of post-graduation entrepreneurship was a significant factor in entrepreneurial success [19]. Scholars have also investigated location of start-up activity amongst Swedish graduates [18] (who were found elsewhere to be unlikely entrepreneurs overall [62]), unveiling patters of geographical clustering. In contrast, our study focused on the relationship between participation in entrepreneurship education in university and post-graduation entrepreneurial activities.

Limitations of this study include those that are intrinsic to our data source. The National Graduate Survey relies on the self-declaration of respondents with respect to their current career status. As well, individual decisions to pursue self-employment and business ownership can also be influenced by several factors such as family background, social, cultural and financial support, and having relevant previous business experience [35, 38, 42, 63, 64]. This suggests that entrepreneurial outcomes may be viewed against a much larger background, against which learning experiences on campus plays a role whose magnitude needs to be better understood in different contexts.

Entrepreneurship education has flourished in postsecondary institutions, and research on the topic is important to gain insight into the type of individuals these programs benefit, both in terms of who enrolls in the programs and with what goals [65] and in terms of post-graduation entrepreneurial outcomes. We provide conclusive evidence that students who join entrepreneurial learning activities on Canadian university campuses are more likely to become self-employed and business owners three years after graduating, but that those outcomes are not similar to all students. We find that the relationship between entrepreneurship education and entrepreneurial outcomes is stronger for men than women. Qualitative and quantitative research that identifies the specific factors deterring women from participating in entrepreneurial learning would provide invaluable insight for program administrators and entrepreneurship educators. Our study also goes beyond previous research by accounting for factors such as race, ethnicity, citizenship, and socio-economic status. Future research might explore the specific mechanisms at play in the relationship between demographic characteristics and entrepreneurial outcomes among university graduates longitudinally.

Our findings are significant to policy makers and educators who are interested in understanding and improving the impacts of entrepreneurship education for university students. Supporters and academic leaders of entrepreneurship education programs in Canadian universities have a clear opportunity to focus on recruiting, retaining, and nurturing students who are underrepresented among entrepreneurs, alongside the effort to stimulate a greater number of young entrepreneurs in general. Efforts to understand and intervene on the factors preventing some groups of university students from considering entrepreneurship is an attractive option might be embedded into government, non-profit, and institutional programs supporting entrepreneurship education in universities. The educational and socialization context of university campuses provides a platform for addressing real and perceived barriers that discourage some groups from considering entrepreneurship as a career option, but this requires deliberate efforts to identify such barriers and act on them. Evidence-based approaches for student recruitment, training, and mentoring that consider their particular views and experiences vis-à-vis entrepreneurship are needed, going beyond common surface-level attempts to represent diversity in entrepreneurial programming [66].
